# Supplementary material for: The Cost-Effectiveness of the SMART Work & Life Intervention for Reducing Sitting Time
Source: Int J Environ Res Public Health. 2022 Nov 11;19(22):14861. doi: 10.3390/ijerph192214861 (PMC9690649; doi:10.3390/ijerph192214861)

Supplementary Appendix

Table S1: Unit costs

Resource	Unit costs	Sources
**Primary care**		
General practitioner (GP) - Surgery visit	£33.00	Personal Social Services Research Unit, 2019
General practitioner (GP) - Home visit	£107.07	Personal Social Services Research Unit, 2010
General practitioner (GP) - Phone call	£15.32	Personal Social Services Research Unit, 2019
General practice Nurse - Surgery visit	£5.69	Personal Social Services Research Unit, 2019
General practice Nurse - Home visit	£31.44	Personal Social Services Research Unit, 2010
General practice Nurse - Phone call	£6.00	Personal Social Services Research Unit, 2019
NHS Walk-in Centre Visit	£46.00	NICE guideline 94 (2018)
NHS Urgent Care Centre Visit	£67.00	NICE guideline 94 (2018)
**Community services**		
Occupational Health Nurse	£39.42	NHS reference costs 2017/2018
**Mental health care**		
Mental health nurse	£92.00	Personal Social Services Research Unit, 2019
Other counsellor/therapist	£182.71	NHS Reference cost 2017/18 [MHSTOTHPLA]
**Secondary care**		
Accident and Emergency Visit	£112.41	Personal Social Services Research Unit, 2010
Hospital outpatient appointments	£137.58	NHS reference costs 2017/2018 [General Surgery]

Table S2: Imputed HRQoL, QALY and absenteeism by treatment arm and follow-up period

	**SWAL-desk**			**SWAL-only**			**Control**		
	**Baseline**	**Month 3**	**Month 12**	**Baseline**	**Month 3**	**Month 12**	**Baseline**	**Month 3**	**Month 12**
EQ-5D-5L	0.90031	0.90788	0.89748	0.89944	0.90484	0.89473	0.90333	0.90272	0.89956
(Mapped) EQ-5D-3L	0.84358	0.85060	0.83568	0.83693	0.84956	0.83969	0.84589	0.84893	0.84260
QALYs (5L)†	-	-	0.89879	-	-	0.89520	-	-	0.89832
QALYs (3L)*†	-	-	0.84144	-	-	0.84113	-	-	0.84712
Absenteeism (days)	0.81917		1.61583	0.95984		1.40884	1.47865		1.06891

† Total within-trial QALYs average imputed values; all other outcomes presented as the average of complete cases
*Calculated from EQ-5D-3L scores mapped from EQ-5D-5L responses

Table S3: Base case QALY regression – ordinary least squares regression

	Coef.	St.Err.	t-value	p-value	[95% Conf	Interval]	Sig
Baseline EQ5D	0.348	0.044	7.91	0.000	0.261	0.435	***
SWAL-only	0.005	0.011	0.4	0.690	-0.018	0.027	
SWAL-desk	-0.001	0.015	-0.03	0.973	-0.032	0.031	
Age	-0.001	0.001	-1.19	0.244	-0.002	0.001	
Female	-0.014	0.012	-1.2	0.240	-0.039	0.010	
Non-white	0.003	0.013	0.25	0.807	-0.023	0.030	
Baseline bmi	-0.002	0.001	-1.98	0.051	-0.004	0.000	*
Salford	-0.002	0.011	-0.15	0.885	-0.023	0.020	
Liverpool	0.004	0.013	0.34	0.731	-0.021	0.030	
Large cluster	0.002	0.012	0.13	0.898	-0.023	0.026	
Constant	0.646	0.054	11.95	0.000	0.539	0.753	***

*** *p<0.01*, ** *p<0.05*, * *p<0.1*

EQ5D: Euroqol 5-level; SWAL: SMART Work & Life; Coef: Coefficient; St.Err: Standard Error; Sig: Significance; bmi: Body mass index

Table S4: Complete case secondary outcomes

Mean (SD)	Baseline value			3 months			12 months			Change at 3 months			Change at 12 months		
	Control	SWAL-only	SWAL-desk	Control	SWAL-only	SWAL-desk	Control	SWAL-only	SWAL-desk	Control	SWAL-only	SWAL-desk	Control	SWAL-only	SWAL-desk
**Stress** [a]	15.9 (6.52)	16.4 (7.02)	16.7 (1.00)	16.0 (6.69)	16.1 (6.55)	15.3 (6.83)	16.1 (6.54)	16.3 (6.61)	15.6 (7.16)	0.43 (5.10)	-0.26 (5.41)	-0.58 (5.64)	0.44 (5.16)	-0.12 (5.55)	-0.24 (5.57)
**Wellbeing** [b]	54.7 (20.1)	54.0 (20.3)	55.4 (19.6)	56.2 (20.6)	57.0 (21.5)	58.9 (20.0)	55.6 (20.6)	55.5 (20.6)	57.8 (20.1)	0.05 (13.9)	2.46 (16.3)	2.37 (17.0)	0.69 (14.5)	2.06 (19.7)	2.12 (15.8)
**Job performance** [c]	5.52 (1.01)	5.54 (1.05)	5.53 (0.93)	5.45 (1.00)	5.47 (1.02)	5.44 (0.99)	5.35 (0.99)	5.45 (1.08)	5.46 (0.98)	0.06 (0.82)	-0.02 (0.96)	-0.11 (0.99)	-0.12 (0.94)	-0.10 (1.03)	-0.06 (1.10)
**Job satisfaction** [d]	4.80 (1.38)	4.97 (1.29)	4.89 (1.24)	4.79 (1.41)	4.98 (1.30)	4.76 (1.30)	4.69 (1.40)	5.01 (1.33)	4.74 (1.29)	-0.02 (0.98)	0.04 (0.94)	-0.10 (1.04)	-0.07 (1.17)	0.01 (1.17)	-0.14 (1.27)
**Work engagement** [e]															
Vigour	3.31 (1.33)	3.42 (1.27)	3.30 (1.25)	3.38 (1.34)	3.52 (1.19)	3.50 (1.19)	3.35 (1.29)	3.59 (1.14)	3.46 (1.17)	0.09 (0.86)	0.10 (0.95)	0.16 (0.87)	0.05 (0.96)	0.17 (0.93)	0.16 (0.98)
Dedication	4.13 (1.22)	4.23 (1.23)	4.09 (1.16)	4.04 (1.14)	4.11 (1.17)	4.10 (1.04)	4.00 (1.18)	4.22 (1.16)	4.06 (1.08)	-0.09 (0.75)	-0.10 (0.77)	0.02 (0.75)	-0.11 (0.85)	-0.05 (0.84)	-0.02 (0.94)
Absorption	4.14 (1.13)	4.22 (1.11)	4.16 (1.06)	4.11 (1.01)	4.15 (0.99)	4.24 (1.01)	4.11 (1.04)	4.21 (1.06)	4.26 (1.06)	0.02 (0.90)	-0.07 (0.82)	0.05 (0.80)	0.06 (0.87)	-0.08 (0.93)	0.08 (0.94)
Overall	3.86 (1.10)	3.96 (1.08)	3.85 (1.01)	3.84 (1.05)	3.93 (1.02)	3.94 (0.97)	3.82 (1.04)	4.00 (1.01)	3.93 (1.00)	0.00 (0.66)	-0.03 (0.67)	0.08 (0.61)	-0.00 (0.73)	0.01 (0.71)	0.07 (0.78)

[a] Stress (Never=0; Very Often=4), score 0-40, with higher score indicating greater stress; [b] Wellbeing No time = 1, all of the time = 5, score 0-100, with a higher score indicating higher wellbeing; [c] Job performance (1 = Very Poorly; 7 = Extremely Well); [d] Job satisfaction (1 = Dissatisfied; 7 = Extremely Satisfied); [e] Work engagement (0 = Never; 6 Always), higher scores indicate greater work engagement

Table S5: Base case absenteeism days regression - generalised linear model (family ~ gamma; link – log)

	Coef.	St.Err.	t-value	p-value	[95% Conf	Interval]	Sig
Baseline absenteeism	0.062	0.04	1.56	0.122	-0.017	0.141	
SWAL-only	0.233	0.398	0.58	0.567	-0.615	1.080	
SWAL-desk	0.401	0.495	0.81	0.433	-0.677	1.480	
Age	0.019	0.014	1.40	0.173	-0.009	0.047	
Female	0.152	0.373	0.41	0.687	-0.612	0.915	
Non-white	0.638	0.388	1.65	0.109	-0.151	1.428	
Baseline bmi	0.027	0.036	0.75	0.47	-0.053	0.107	
Salford	-0.016	0.339	-0.05	0.963	-0.686	0.654	
Liverpool	-0.213	0.596	-0.36	0.728	-1.517	1.092	
Large cluster	0.334	0.430	0.78	0.453	-0.611	1.279	
Constant	-2.188	1.141	-1.92	0.08	-4.681	0.305	*

*** $p<0.01$, ** $p<0.05$, * $p<0.1$

SWAL: SMART Work & Life; Coef: Coefficient; St.Err: Standard Error; Sig: Significance; bmi: Body mass index

Table S6: Intervention costs

	Costs	
	Trial total	Per ITT individual
**SMART Work & Life Plus Desk (n=240)**		
Direct costs of providing workplace champion training[1]	£1,776.00	£7.40
Participant time to engage with components costs[2]	£13,076.91	£54.49
Workplace champions' training time costs	£1,419.84	£5.92
Workplace champions' facilitation time costs	£3,590.40	£14.96
Correspondence to managers	£145.19	£0.60
Desk purchases	£34,041.79	£141.84
Desk set-up labour costs	£720.00	£3.00
Motivational materials	£25.00	£0.10
Total	£54,795.13	£228.31
**SMART Work & Life (n=249)**		
Direct costs of providing workplace champion training training[1]	£1,776.00	£7.13
Participant time to engage with components costs[2]	£12,377.61	£49.71
Workplace champions training time costs	£1,321.92	£5.31
Workplace champions' facilitation time costs	£4,412.93	£17.72
Correspondence to managers	£151.03	£0.61
Motivational materials	£27	£0.11
Total	£20,066.49	£80.59

1 Planning and preparation training session, travel, delivery of training

2 Online education sessions; reading monthly emails; installation and set-up of self-monitoring apps/software; sitting less challenges and coaching sessions

Table S7: Complete case within-trial costs by treatment arm and resource category

	SWAL-desk			SWAL-only			Control		
	N	Mean (SD)	Min, Max	N	Mean (SD)	Min, Max	N	Mean (SD)	Min, Max
**Complete case**									
Primary care									
GP surgery visit	136	71.82 (90)	(0,528)	125	96.62 (251)	(0,2574)	116	87.05 (130)	(0,726)
GP home visit	136	0.00 (0)	(0,0)	125	0.00 (0)	(0,0)	116	0.00 (0)	(0,0)
GP phone call	136	9.01 (24)	(0,153)	125	6.86 (18)	(0,123)	116	9.77 (29)	(0,214)
GP nurse surgery visit	136	6.02 (13)	(0,114)	125	5.37 (10)	(0,57)	116	4.91 (9)	(0,46)
GP nurse visit	136	0.46 (5)	(0,63)	125	0.00 (0)	(0,0)	116	0.00 (0)	(0,0)
GP nurse phone call	136	0.35 (2)	(0,12)	125	0.1 (1)	(0,12)	116	0.21 (2)	(0,12)
NHS walk-in centre visit	136	2.71 (19)	(0,184)	125	12.51 (41)	(0,276)	116	6.34 (23)	(0,92)
NHS urgent care centre visit	136	1.97 (16)	(0,134)	125	5.36 (26)	(0,134)	116	3.47 (21)	(0,134)
Community services									
Occupational Health Nurse	136	2.90 (28)	(0,315)	125	4.42 (31)	(0,237)	116	6.80 (40)	(0,394)
Mental health care									
Mental health nurse	136	2.71 (22)	(0,184)	125	1.47 (16)	(0,184)	116	1.59 (17)	(0,184)
Other counsellor/therapist	136	150.47 (684)	(0,5481)	125	242.64 (835)	(0,7308)	116	207.91 (631)	(0,4385)
Secondary care									
Accident and Emergency Visit	136	14.88 (62)	(0,450)	125	8.99 (53)	(0,450)	116	15.50 (64)	(0,450)
Outpatient appointments	136	153.77 (343)	(0,2476)	125	123.27 (289)	(0,1926)	116	142.32 (247)	(0,1101)
Inpatient days	136	0 (0)	(0,0)	125	65.59 (733)	(0,8199)	116	11.76 (89)	(0,682)
Total									
Total health-related costs	136	417.07 (825)	(0,5481)	125	573.21 (1425)	(0,11805)	116	497.63 (839)	(0,5837)

Table S8: Base case health resource cost regression – generalised linear model (family ~ gamma; link – log)

	Coef.	St.Err.	t-value	p-value	[95% Conf	Interval]	Sig
SWAL-only	-0.054	0.141	-0.39	0.700	-0.333	0.224	
SWAL-desk	-0.215	0.147	-1.46	0.147	-0.507	0.077	
Age	-0.010	0.006	-1.59	0.117	-0.023	0.003	
Female	0.416	0.157	2.65	0.011	0.100	0.732	**
Non-white	-0.072	0.168	-0.43	0.672	-0.414	0.271	
Baseline bmi	0.034	0.012	2.86	0.006	0.010	0.058	***
Salford	-0.076	0.150	-0.50	0.615	-0.372	0.221	
Liverpool	-0.392	0.233	-1.68	0.100	-0.861	0.077	*
Large cluster	0.280	0.141	1.99	0.051	-0.002	0.562	*
Constant	5.647	0.381	14.82	0.000	4.894	6.400	***

*** $p<0.01$, ** $p<0.05$, * $p<0.1$

SWAL: SMART Work & Life; Coef: Coefficient; St.Err: Standard Error; Sig: Significance; bmi: Body mass index

Table S9: Model parameters

	Base case values	PSA distributional form			Source
**Cohort characteristics**		Distribution	Mean	SD	
Sex (male)	27.65%	N/A			SMART Work & Life trial
Age (at extrapolation)	41	N/A			SMART Work & Life trial
Baseline sedentary time (minutes/day)	605.24 minutes	Normal	605.2	82.2	SMART Work & Life trial
**Treatment effect**					
SWAL-desk (reduction in sedentary time)	63.7 minutes	Normal	63.7	8.34	SMART Work & Life trial
SWAL-only (reduction in sedentary time)	22.2 minutes	Normal	22.2	8.44	SMART Work & Life trial
Annual treatment-effect decay rate	50%	-	-	-	Assumed in prior works (37,38,48)
Sedentary associated all-cause hazards	See Figure 1	Normal	See Figure 1		Ekelund et al (16)
**Discount rate**					
Costs	3.5%	N/A			NICE Methods Guide (20)
QALYs	3.5%	N/A			NICE Methods Guide (20)

Figure S1: Ekeland et al interpolated hazard ratios

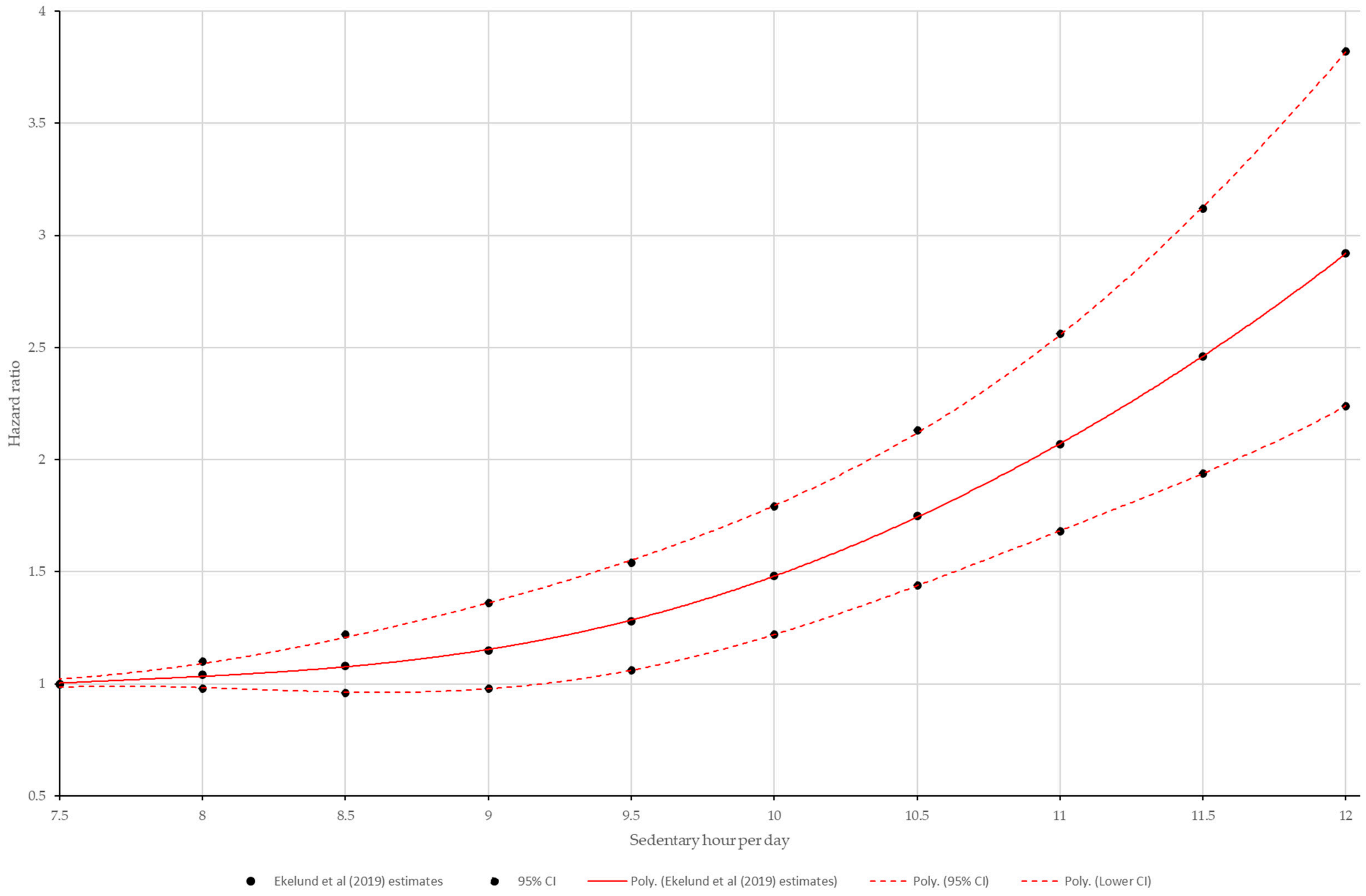

Figure S2: Sedentary time modelled for each comparator

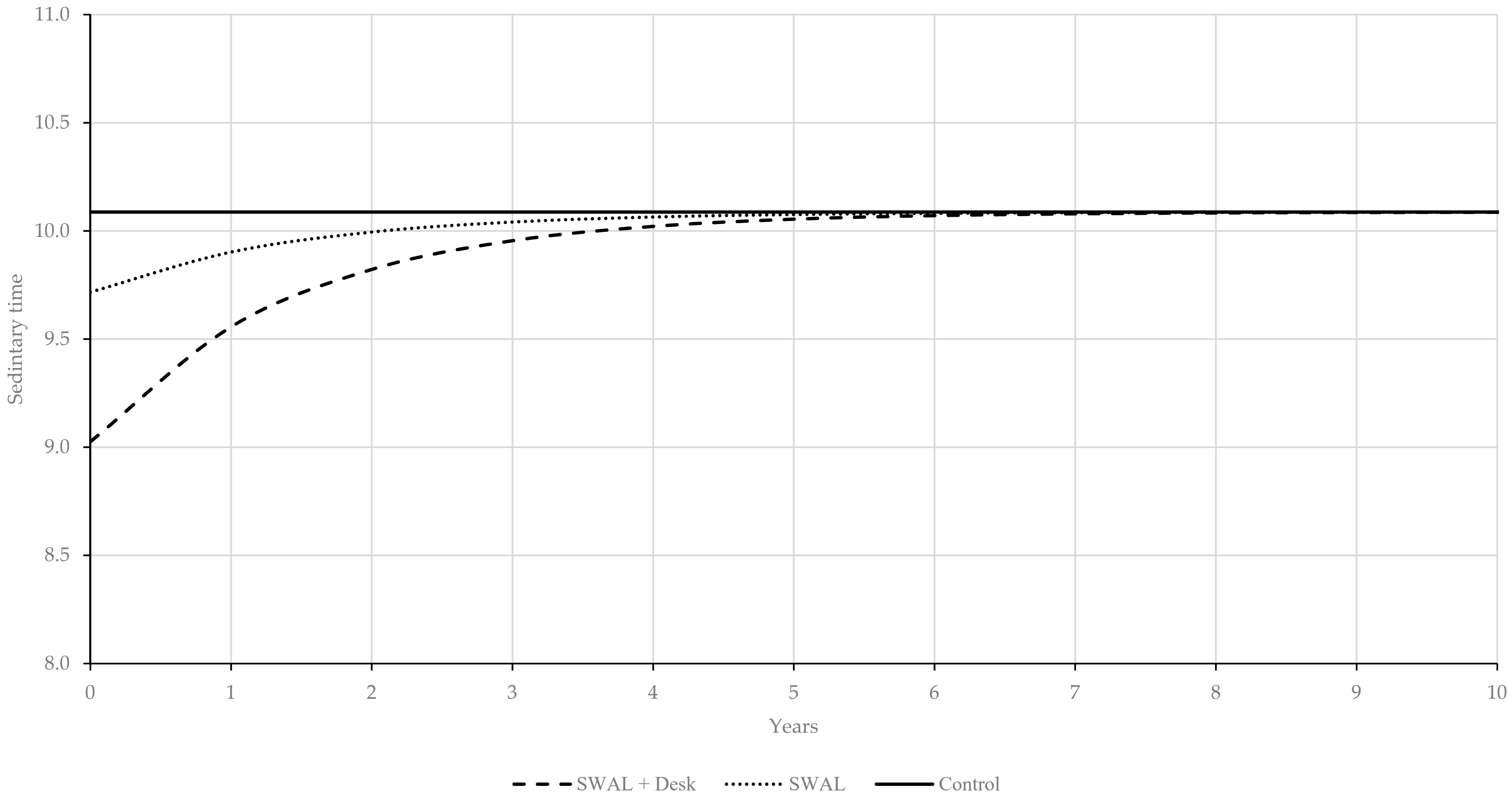

Figure S3: Hazard ratios associated with the sedentary time modelled for each comparator

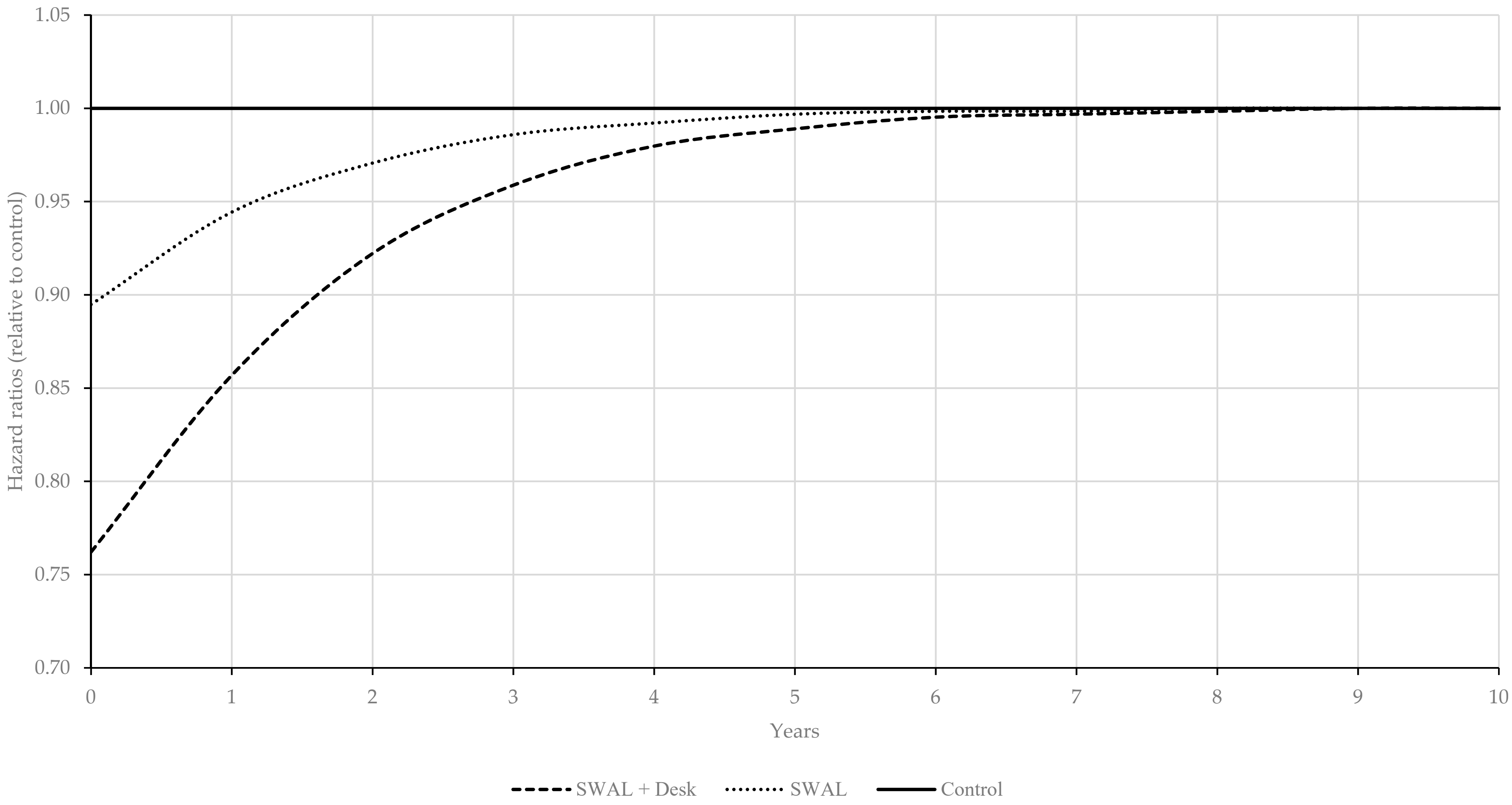

Figure S4: Incremental net health benefits for alternative age and treatment decay profiles at a cost-effectiveness threshold of £20,000 per QALY

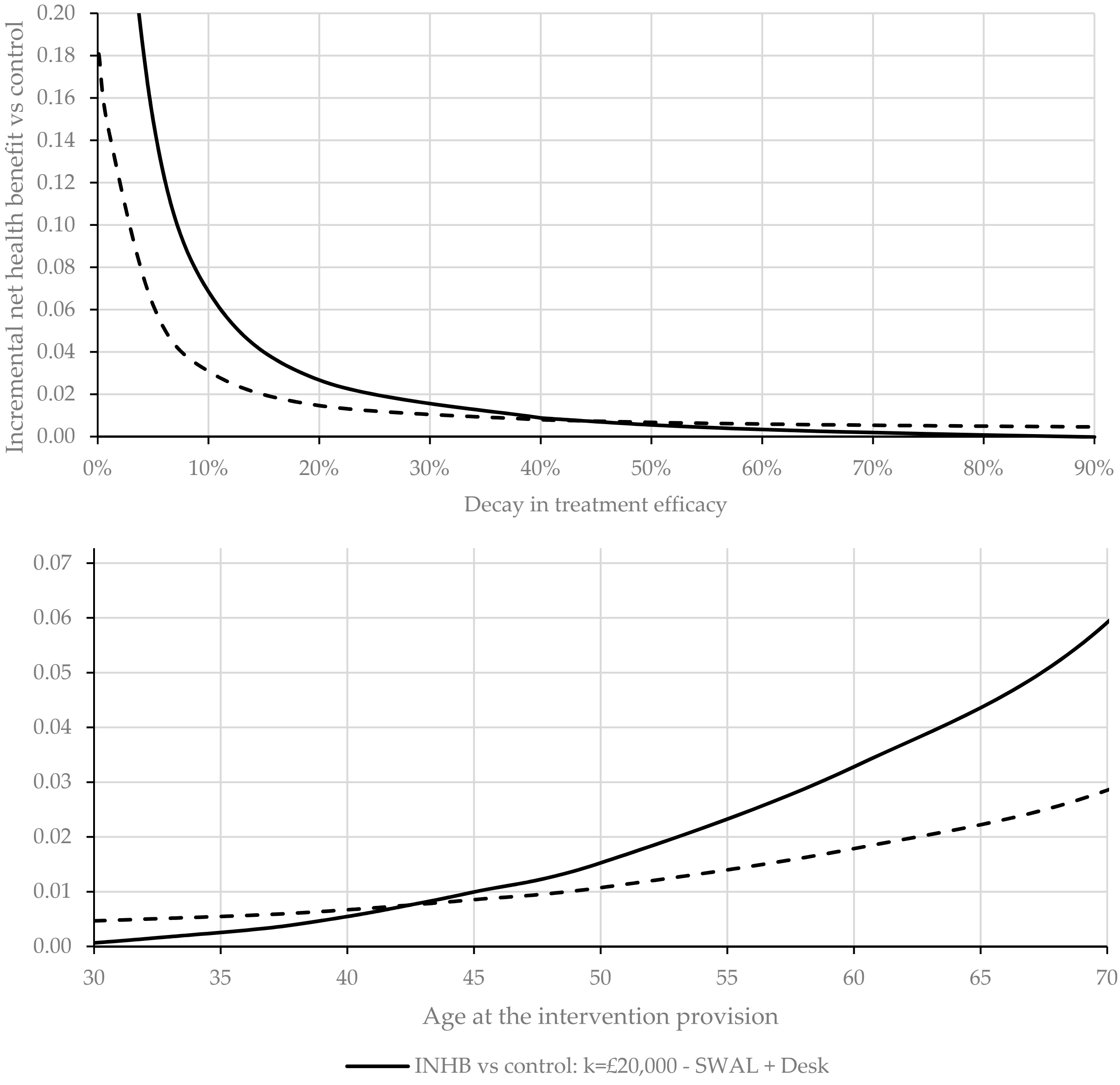

Figure S5: Incremental net health benefits for alternative age and treatment decay profiles at a cost-effectiveness threshold of £30,000 per QALY

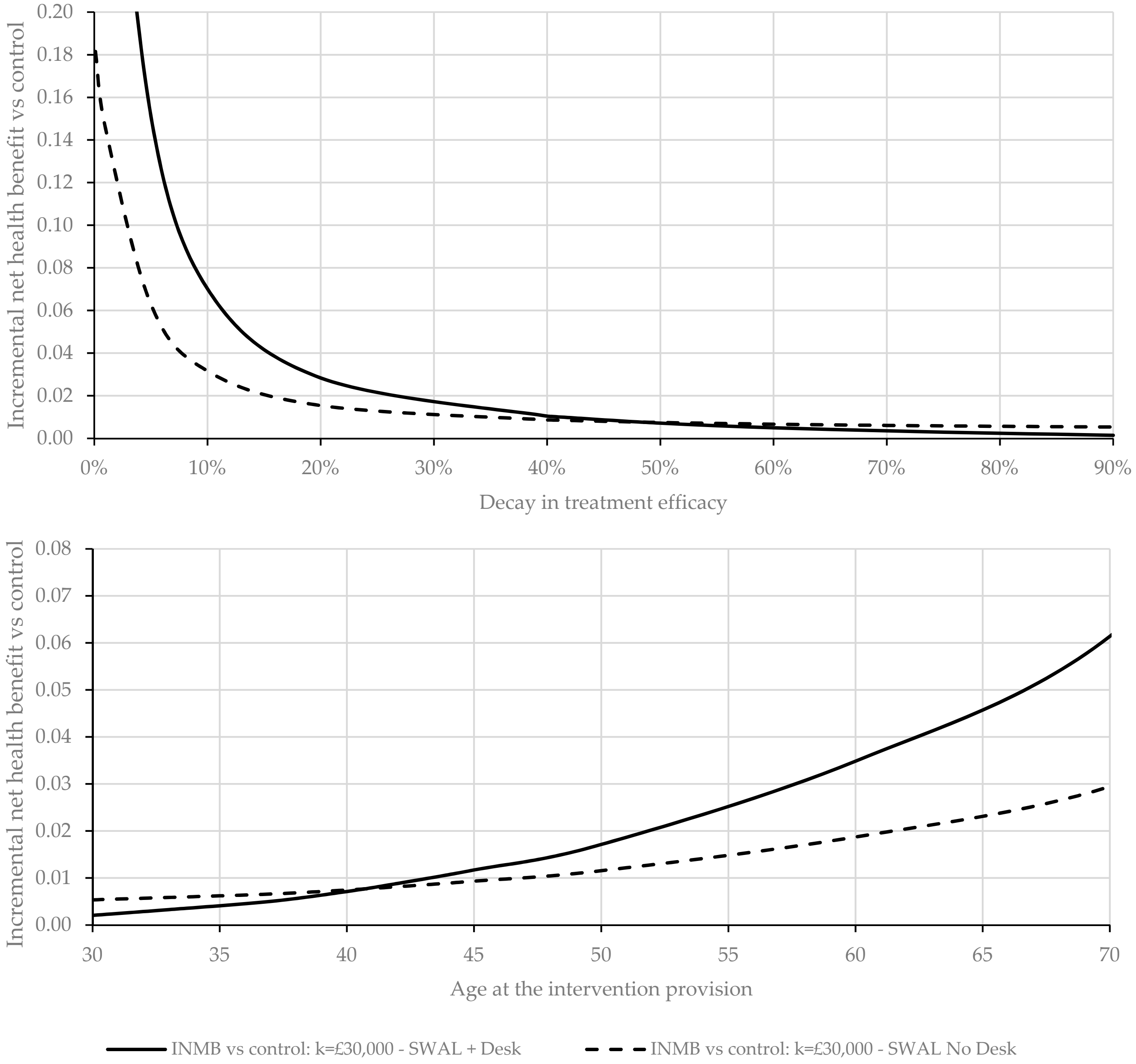

Supplement: Supplementary file 1 [file ijerph-19-14861-s001.zip › ijerph-1998386-supplementary.pdf]
